# Reconsidering Electrocardiographic Predictors of Culprit Coronary Artery Occlusion in NSTEMI Patients

**DOI:** 10.1111/anec.70112

**Published:** 2025-09-10

**Authors:** Mucahit Yetim, Ömer Burak Çelik, Macit Kalçık

**Affiliations:** ^1^ Department of Cardiology, Faculty of Medicine Hitit University Corum Turkey

**Keywords:** acute total occlusion, electrocardiography, left circumflex artery, non‐ST‐elevation myocardial infarction (NSTEMI)

## Abstract

This letter provides a critical appraisal of the study by Wei et al. on clinical and electrocardiographic predictors of left circumflex artery occlusion in NSTEMI patients. While the authors identified STV5 + STV6 ≥ 2.5 mm and T‐wave imbalance as potential markers, concerns remain regarding the single‐center, retrospective design, limited sensitivity of ECG findings, and the lack of significant differences in clinical outcomes. Prior meta‐analyses suggest a higher risk in patients with occluded culprit arteries, highlighting inconsistencies with the present study. Future research should employ multicenter prospective designs and advanced diagnostic modalities, including posterior ECG leads and artificial intelligence–based analysis, to improve detection and risk stratification of culprit LCX occlusion in NSTEMI.

## Author Contributions

M.Y.: writing, Ö.B.Ç.: editing, M.K.: editing and review.

## Conflicts of Interest

The authors declare no conflicts of interest.

To the Editor,

1

We read with great interest the article by Wei et al. (2025) entitled “Clinical and Electrocardiographic Characteristics in NSTEMI Patients With Acute Total Occlusion of Culprit Left Circumflex Artery” published in the *Annals of Noninvasive Electrocardiology*. The authors provide important insights into the clinical features and electrocardiographic markers associated with left circumflex artery (LCX) occlusion in NSTEMI patients. However, we would like to raise several critical points regarding study design, interpretation, and clinical applicability.

First, the retrospective and single‐center nature of the study raises concerns about external validity. Previous investigations have shown that occlusive culprit lesions in NSTEMI vary significantly across populations and healthcare systems, suggesting that multicenter and prospective studies are required to avoid selection bias (Hung et al. 2018). Without broader data, the generalizability of the reported predictors such as STV5 + STV6 ≥ 2.5 mm and T‐wave imbalance remains limited.

Second, the study reports no significant differences in major adverse cardiac events (MACEs) between LCX and other culprit vessel groups, despite higher biomarker levels and reduced ejection fraction in LCX patients. This contrasts with prior meta‐analyses demonstrating that NSTEMI patients with an occluded culprit artery often have worse outcomes compared to those with non‐occlusive lesions (Khan et al. 2017). The discrepancy may result from underpowered subgroup analyses and relatively short follow‐up duration.

Third, the electrocardiographic predictors identified in this study suffer from limited sensitivity, reducing their utility in real‐world emergency care. Earlier work emphasized that ECG findings in NSTEMI are dynamic and strongly influenced by factors such as collateral circulation and thrombus resolution (Figueras et al. 2018). Hence, relying solely on static ECG markers may risk underdiagnosis or misclassification of high‐risk patients.

Finally, future research should integrate advanced diagnostic modalities such as posterior ECG leads, high‐resolution imaging, and artificial intelligence–based algorithms to improve the detection of LCX occlusion in NSTEMI (Dzikowicz and Carey 2023). A multimodal approach could refine risk stratification and optimize revascularization strategies beyond conventional 12‐lead ECG interpretation.

In conclusion, while Wei et al. (2025) make a valuable contribution by highlighting potential ECG predictors of LCX occlusion in NSTEMI, the limitations regarding study design, statistical power, and diagnostic sensitivity should be addressed in future large‐scale studies to ensure robust clinical applicability.

Sincerely,

## Data Availability

The authors have nothing to report.
